# GPR124 Alleviates Blood–Brain Barrier Disruption by Enhancing Microvascular Endothelial Function after Traumatic Brain Injury

**DOI:** 10.1002/advs.202501197

**Published:** 2026-06-15

**Authors:** Chen Wang, Lin Cai, Qiuyuan Gong, Yang Yang, Yuqing Liang, Xinyu Niu, Lai Wei, Ze Liu, Shengju Wu, Yinghui Men, Yaohui Tang, Jun Ding, Hengli Tian, Hao Chen

**Affiliations:** ^1^ Department of Neurosurgery Shanghai Sixth People's Hospital Affiliated to Shanghai Jiao Tong University School of Medicine Shanghai China; ^2^ School of Biomedical Engineering Shanghai Jiao Tong University Shanghai China

**Keywords:** blood–brain barrier, endothelial cells, GPR124, traumatic brain injury, Wnt/β‐catenin signaling

## Abstract

Traumatic brain injury (TBI) is a leading cause of death among young adults worldwide. However, the role of G protein‐coupled receptor 124 (GPR124), a key regulator of the nervous system, in TBI remains unexplored. We employed a controlled cortical impact (CCI) model combined with single‐cell RNA sequencing to analyze the localization of GPR124 expression following TBI. Additionally, we used mice with endothelial cell (EC)‐specific conditional knockout (CKO) of GPR124 to perform behavioral experiments. A stretch injury (SI) model was also established to investigate the effects of GPR124 on ECs. Neurological recovery after TBI was significantly impaired in mice with the EC‐specific CKO of GPR124. Furthermore, GPR124 knockdown reduced EC function after SI. Notably, tight junction integrity was disrupted both in vivo and in vitro after GPR124 knockdown. Mass spectrometry and immunoprecipitation analyses confirmed that GPR124 interacts with fibroblast growth factor binding protein‐1, thereby activating the Wnt/β‐catenin pathway. Our study demonstrates that GPR124 regulates microvascular endothelial function and maintains blood–brain barrier integrity by activating the Wnt/β‐catenin pathway. This mechanism plays a crucial role in improving TBI prognosis and may represent a potential new therapeutic target.

## Graphical abstract

Our study reveals the protective role of GPR124 in maintaining BBB integrity and promoting neurological recovery following TBI. It makes a significant contribution by uncovering a novel molecular interaction between GPR124 and FGFBP1 and linking this to activation of the Wnt/β‐catenin signaling pathway in vascular repair mechanisms.

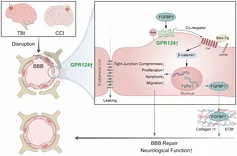

## 1 Introduction

Traumatic brain injury (TBI) remains a major condition threatening human health and poses an increasingly serious global social and public health challenge that has drawn considerable attention internationally [1, 2, 3]. It is widely recognized as a leading cause of death in young adults worldwide [4, 5].

The clinical course of TBI involves two phases: primary injury, which refers to the immediate damage to brain parenchyma resulting from mechanical impact, and secondary injury, which arises from a cascade of cellular and biochemical events, including inflammation, oxidative stress, mitochondrial dysfunction, and apoptosis [6]. In contrast to primary injury, secondary injury begins within minutes after TBI and can persist for months to even years. While little can be done to reverse primary injury, a range of therapeutic interventions targeting secondary injury are possible and have the potential to improve the prognosis of patients with TBI. Therefore, secondary injury represents a critical target in the treatment of TBI. During secondary injury, various pathological processes interact, among which the integrity of the blood–brain barrier (BBB)—the interface between brain tissue and the systemic circulation—is of particular importance. Disruption of the BBB can lead to severe complications, most notably progressive hemorrhagic injury (PHI) [7]. Therefore, restoring BBB integrity after TBI is of clinical significance. However, the damage and repair mechanisms of the BBB are poorly understood and warrant further investigation [8].

G protein‐coupled receptor 124 (GPR124) is a key member of the G protein‐coupled receptor family and plays a critical role in vascular development [9]. Studies have shown that during normal mouse embryonic development, GPR124 is expressed in vascular tissues across multiple systems, including the central nervous system (CNS), where it regulates neural tube angiogenesis and maintains BBB integrity [9, 10]. Anderson et al. found that after global or endothelial‐specific knockout of GPR124 in mice, knockout mice exhibited “glomeruloid” vascular abnormalities in the forebrain and spinal cord regions, thus observing obstructed central vascular invasion and impaired BBB development [11].

The classic Wnt/β‐catenin signaling pathway is a key signaling axis regulating embryonic development, organogenesis, and cell fate determination. In the absence of ligand stimulation, cytoplasmic β‐catenin is continuously phosphorylated and degraded by a disruptive complex composed of Axin, APC, CK1, and GSK3β. However, when the Wnt ligand binds to the receptor Frizzled (FZD) and its co‐receptors LRP5/6, this complex is inhibited, β‐catenin stabilizes and translocates to the nucleus, binds to TCF/LEF transcription factors, and initiates the expression of downstream target genes, thereby regulating cell proliferation, differentiation, and adhesion [12].

In the vascular endothelial system, the Wnt/β‐catenin signaling pathway is crucial for endothelial cell (EC) specialization and homeostasis. This pathway can induce the expression of tight junction proteins and transport‐related molecules, thereby promoting the formation and maintenance of the vascular barrier [13]. In CNS development, Wnt7a/Wnt7b, derived from neural progenitor cells, activates β‐catenin signaling in brain endothelial cells, driving vascular invasion and the establishment of the BBB [14]. Furthermore, the co‐receptors GPR124 and RECK can specifically enhance Wnt7a/Wnt7b signaling; their deficiency leads to phenotypes such as impaired central angiogenesis, BBB developmental defects, and neural tube hemorrhage [10] Therefore, the Wnt/β‐catenin pathway is considered one of the core molecular mechanisms for maintaining endothelial function and regulating the integrity of the BBB. However, the role and underlying mechanism of GPR124 in TBI remain unclear.

In this study, we aimed to evaluate neurological outcomes following TBI in mice with EC‐sconditional knockout (CKO) of GPR124. Additionally, we used small interfering RNA (siRNA) targeting GPR124 to suppress its expression and assess its impact on the biological functions of vascular ECs. Finally, we explored the mechanism by which GPR124 exerts its effects, focusing on the activation of the Wnt/β‐catenin signaling pathway.

Our findings suggest that GPR124 attenuates secondary injury after TBI and improves functional recovery by enhancing EC function and restoring BBB integrity, identifying GPR124 as a potential therapeutic target for TBI.

## 2 Results

### 2.1 Characterization of GPR124 Expression after TBI

To investigate changes in GPR124 expression following TBI, we first performed immunohistochemistry (IHC) on clinical samples. Four cases were collected for analysis. Surgical specimens from three patients with intracerebral hemorrhage (ICH) were used as controls. IHC results showed increased GPR124 expression after TBI (Figure S1A). To confirm this upregulation, we conducted western blotting (WB) (Figure S1B,C) and quantitative reverse transcription polymerase chain reaction (qRT‐PCR) (Figure S1D) using fresh frozen samples. These results indicated that, compared with the ICH group, both protein and mRNA levels were significantly higher in the TBI group. In the absence of trauma, intracranial GPR124 expression was extremely low or undetectable (Figure S1E). A controlled cortical impact (CCI) model was established in C57BL/6 mice in the early stage of the study (Figure S1F). To investigate temporal changes in GPR124 expression, brain tissues were collected from the mice at 1, 3, 5, 7, and 14 d post‐TBI. WB (Figure S1G,H) and qRT‐PCR (Figure S1I) results indicated that GPR124 expression in the TBI group was higher than that in the sham group, peaking between 3 and 5 d and remaining high through 14 days. These findings suggest that TBI in adulthood induces early and sustained upregulation of GPR124.

### 2.2 Single‐Cell Sequencing Analysis Reveals that GPR124 is Primarily Expressed in Vascular ECs

**FIGURE 1:**
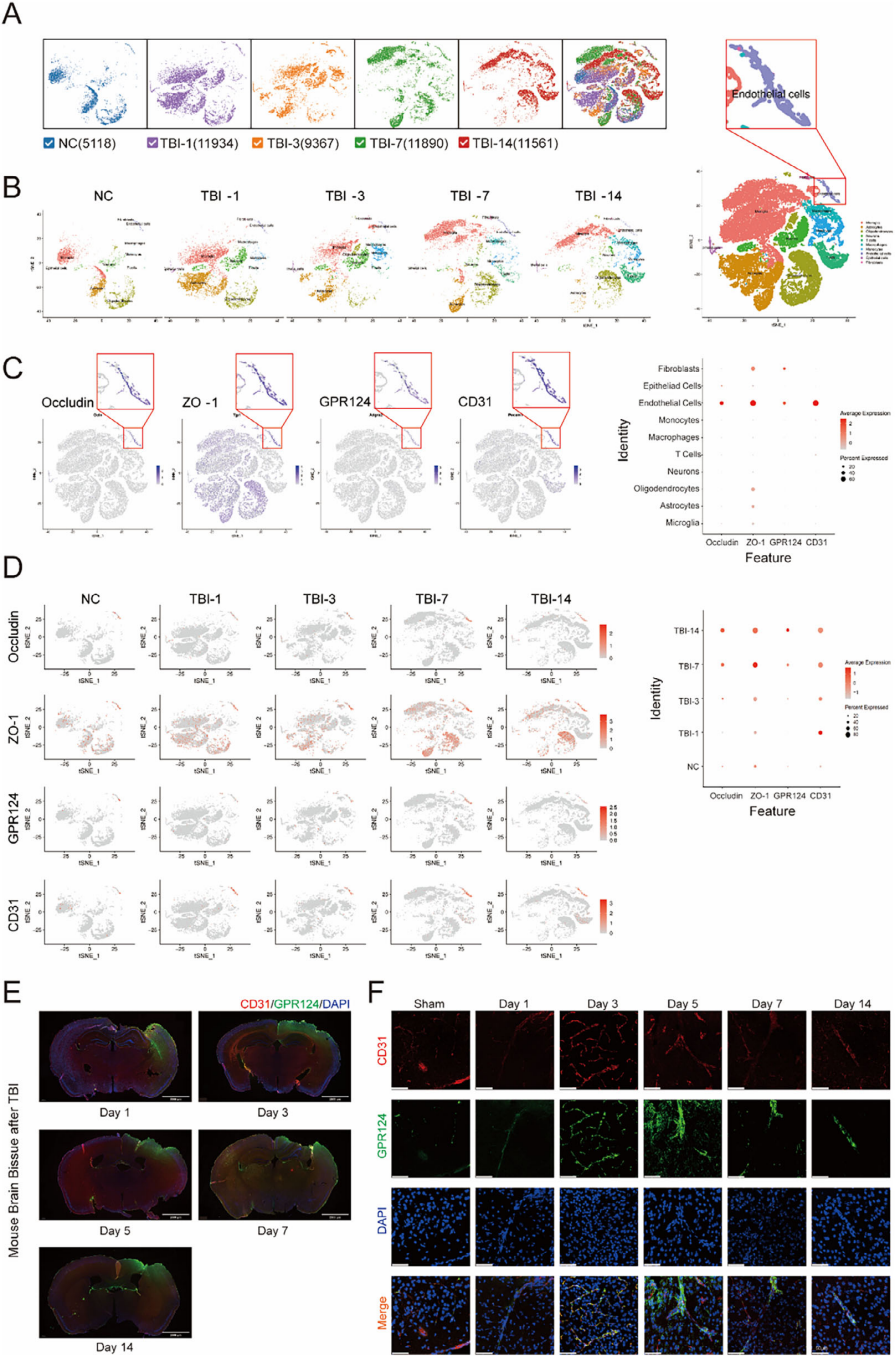
Single‐cell sequencing analysis and IHF of the mouse CCI model showed that GPR124 was mainly expressed on vascular endothelial cells. (A) Total number of single‐cell after‐tissue dissociation of cortical regions in mouse brain tissue from NC, and TBI groups at 1, 3, 5, 7, and 14 days post‐insult. (B) Identification of cell types in mouse brain tissue from NC, and TBI groups at 1, 3, 5, 7, and 14 days post‐insult. (C) Comparison analysis results of CD31^+^, Occludin^+^, ZO‐1^+^ cell subtypes and GPR124+ cell subtypes in the single‐cell sequencing analysis results of the mouse CCI model. (D) Expression abundance results of CD31, occludin, ZO‐1, and GPR124 under different time points in the mouse CCI model. The co‐localization of GPR124 and blood vessels was further confirmed. (E,F) Representative fluorescence images showing dual staining of GPR124(green) and endothelial cell ‐specific biomarker CD31(red) in mouse brain tissue from TBI groups at 1, 3, 5, 7, and 14 days post‐insult. Cell nuclei are shown in blue with DAPI. Scale bar, 2000 µm for (E) and 50 µm for (F).

Using our established single‐cell RNA sequencing database (Figure 1A,B), we observed increased GPR124 expression colocalized with EC markers occludin, ZO‐1, and CD31 (Figure 1C,D). To confirm its localization, we co‐stained GPR124 with CD31 in mouse brain tissue from sham and TBI groups collected at various time points. GPR124 was predominantly expressed in the peri‐contusion region (Figure 1E) and co‐localized with CD31 (Figure 1F), indicating its presence in vascular ECs. Additionally, dual staining with neuronal marker NeuN and microglial marker IBA revealed no co‐localization with either protein (Figure S2A,B).

Collectively, these findings suggest that GPR124 is selectively expressed in vascular ECs following TBI.

### 2.3 Knocking out GPR124 Aggravates Neurological Deficits Following TBI

**FIGURE 2:**
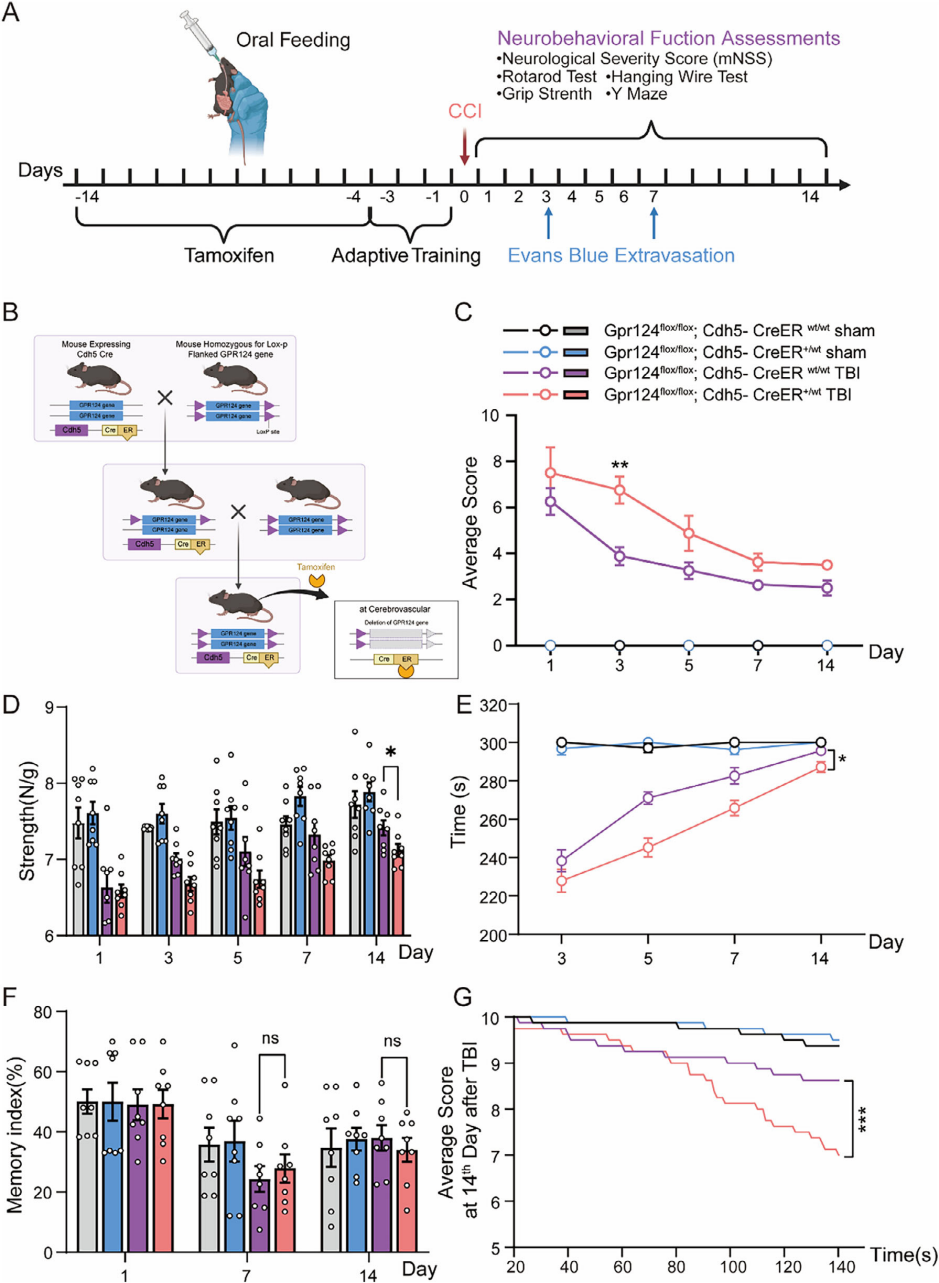
GPR124 attenuated neurological impairment and promoted neurobehavioral recovery in mice CCI models. (A) Overview of experimental timeline for in vivo studies and neurobehavioral tests. (B) The principle of EC‐specificGPR124 conditional knockout (CKO). This study used CRISPR/Cas9 technology to conditional knock in the 2A‐CreERT2‐WPRE‐polyA expression frame at the stop codon site of the Cdh5 gene by homologous recombination. The Cdh5 gene is expressed in endothelial cells. After the flox site is introduced into the GPR124 gene and tamoxifen is given, tamoxifen binds to the ER receptor, activating the CRE enzyme, and finally specifically removes the GPR124 gene on endothelial cells. For the negative control, CreER allele‐deficient (Gpr124^flox/flox^; Cdh5‐CreER^wt/wt^) offspring mice were selected. (C–G) Graphs showed five neurobehavioral function assessments at indicated time points including mNSS scores (C), Grid‐strength test (D), Rotarod test (E), Hanging Wire test (F), and Y maze test (G). n = 8/group. Gpr124^flox/flox^; Cdh5‐CreER^+/wt^ mice (termed CKO mice) vs. Gpr124^flox/flox^; Cdh5‐CreER^wt/wt^ mice (termed control mice) Data were presented as mean ± SEMs. Student's t test. ***p < 0.001; **p < 0.01; *p < 0.05 vs. control mice.

To investigate the functional role of GPR124 after TBI, we generated EC‐specific GPR124 CKO mice. The protocol is detailed in the Methods section (Figure 2A,B). After 3 days of adaptive training, we assessed neurobehavioral recovery in mice after TBI. CKO mice exhibited more severe neurobehavioral deficits than the sham group (Figure 2C–G). Specifically, GPR124 CKO mice performed poorly on the modified Neurological Severity Score (mNSS) (*p* < 0.05, Figure 2C), grip strength (*p* < 0.05, Figure 2D), rotarod (*p* < 0.05, Figure 2E), Y‐maze (Figure 2F), and hanging wire (*p* < 0.05, Figure 2G) tests. These results suggest that GPR124 contributes to neurological recovery following TBI.

### 2.4 Knockout of GPR124 Leads to More Severe BBB Disruption Following TBI

Previous experiments demonstrated a protective role for GPR124 in neurological function after TBI; however, the underlying mechanisms remained unclear. Previous studies have indicated that GPR124 plays a critical role in maintaining BBB stability and improving the prognosis of various diseases [15]. We hypothesized that GPR124 may confer similar protection in TBI through comparable mechanisms.

**FIGURE 3:**
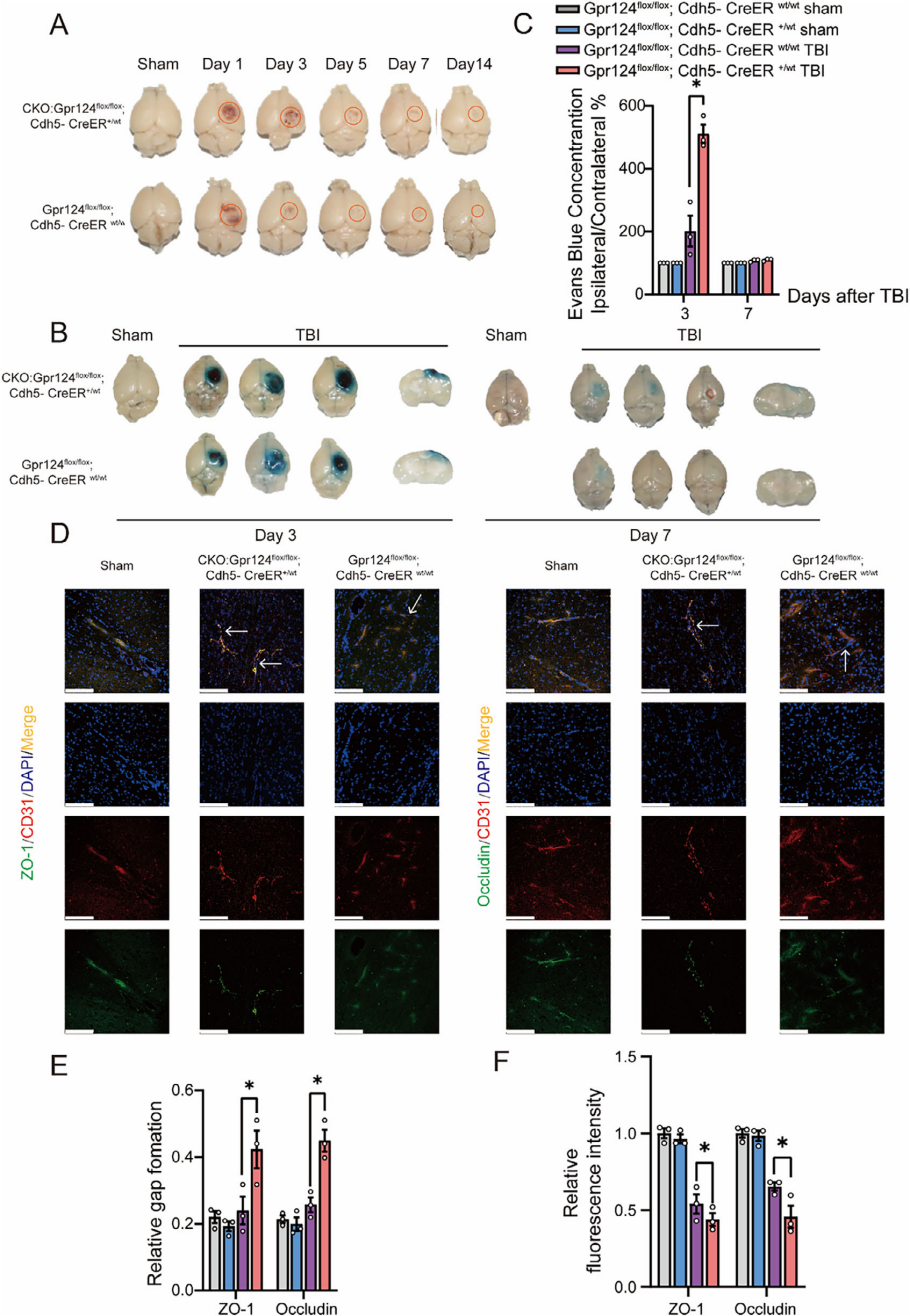
Elevation of GPR124 can alleviate BBB disruption. (A) Comparison of cortical tissue damage in mice at different time points after CCI modeling. The circles represented the approximate area of the lesion cortex. (B) Representative images of EB extravasation in four groups at the indicated time points post‐insult (blue areas indicated the dye extravasation). (C) Statistical results of Figure B. n = 3/group. (D) Representative fluorescence images of dual staining of tight junction protein ZO‐1 (green), tight junction protein Occludin (green), and EC biomarker CD31 (red) in mice brain tissue of TBI at 3 days post‐insult and control groups. Cell nuclei were shown in blue (DAPI). Scale bar, 50 µm. (E) Image J software was used to calculate and analyze the ratio of the gap between tight junction proteins ZO‐1 and Occludin to the total length of blood vessels. (F) Image J software was used to calculate and analyze the relative fluorescence intensity of ZO‐1 and Occludin in the four groups. Data were presented as mean ± SEMs. Student's t test. ***p < 0.001; **p < 0.01; *p < 0.05, Gpr124^flox/flox^; Cdh5‐CreER^+/wt^ mice (termed CKO mice) vs. Gpr124^flox/flox^; Cdh5‐CreER^wt/wt^ mice (termed control mice).

First, the amount of extravasated blood was significantly lower in the control group than that in the CKO group at 1 and 3 days post‐TBI (Figure 3A). We performed an Evans blue (EB) extravasation assay to evaluate the protective function of GPR124 against BBB disruption. Compared with the control group, the EB leakage in the ipsilateral cortex was higher in the CKO group at 3 d after TBI, whereas no significant difference was observed at 7 days (*p* < 0.05, Figure 3B,C). These findings suggest that GPR124 deficiency exacerbates vascular leakage after TBI, indicating that GPR124 may help attenuate BBB disruption.

Furthermore, we performed IHC of tight junction proteins and CD31 in CKO and control mice at 3 days post‐CCI. Compared with the control group, the CKO group exhibited reduced continuity of tight junction proteins in the BBB (Figure 3D). We then quantified the ratio of gaps between tight junction proteins ZO‐1 and occludin relative to total blood vessel length, finding a significantly higher gap ratio in the CKO group at 3 d post‐TBI (Figure 3E). Additionally, quantitative analysis of immunofluorescence intensity showed that ZO‐1 and occludin expression was significantly lower in the CKO group than that in the control group (*p* < 0.05; Figure 3F). Taken together, these findings suggest that GPR124 helps maintain BBB stability by regulating the expression of tight junction proteins.

### 2.5 Knockdown of GPR124 Impairs EC Function Following In Vitro Stretch Injury

**FIGURE 4:**
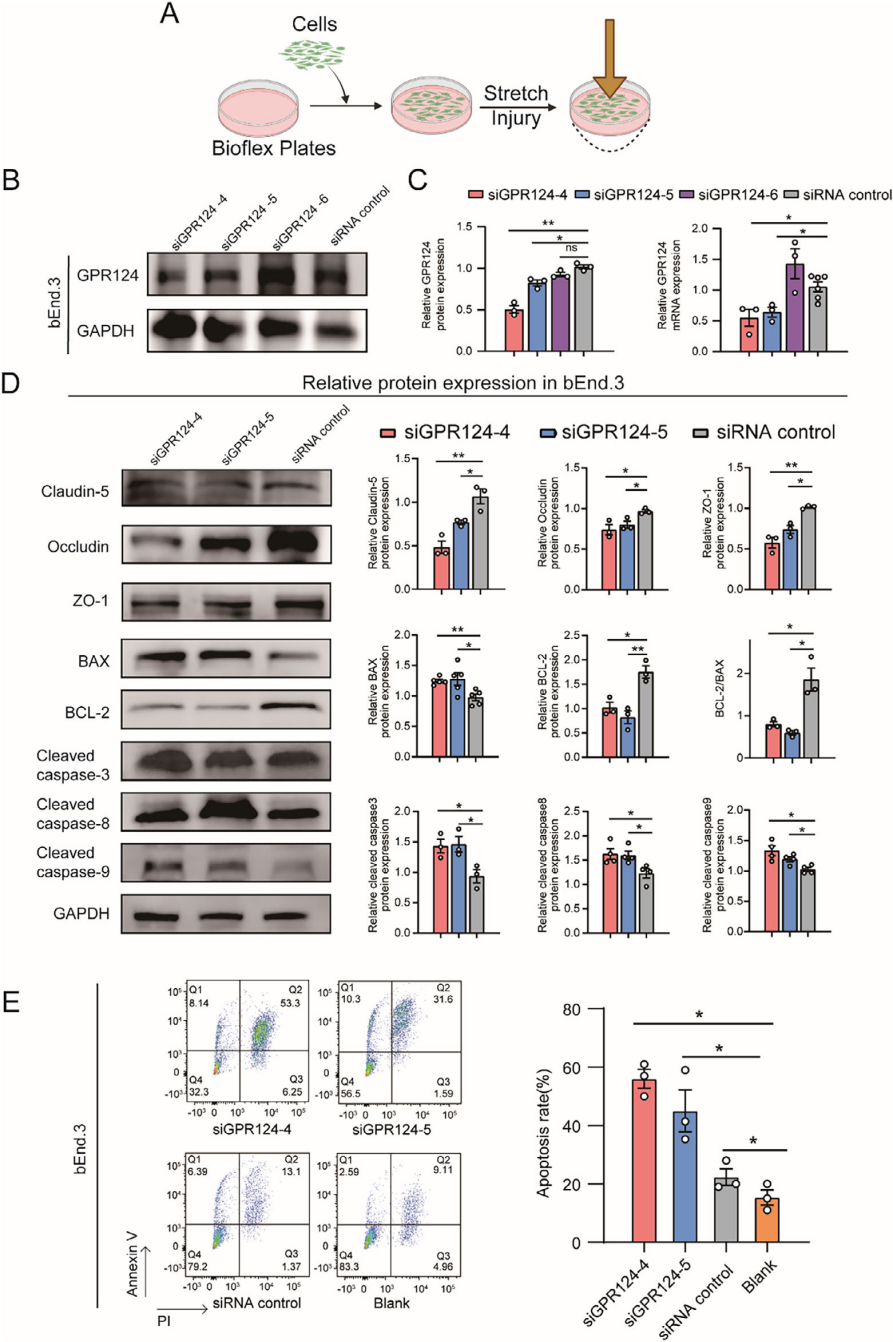
GPR124 protected tight junctions and decreased apoptosis of bEnd.3 cells in vitro. (A) Schematic diagram of the cell stretch injury model. After the cells were planted on the BioFlex plate for 24 h, they were shocked with nitrogen gas of the same force, causing the plate to deform in the same way, and the cells were stretched. (B) Representative western blot results of GPR124 and GAPDH in bEnd.3 cell after stretch injury transfected with siRNAs. (C) Quantitative analysis of western blot results of GPR124 in bEnd.3 cell after stretch injury transfected with siRNAs. GAPDH was used as the control for protein loading. qRT‐ PCR analysis of GPR124 mRNA in bEnd.3. n = 3/group. (D) Representative western blot results and quantitative analysis of claudin‐5, occludin, ZO‐1, BAX, BCL‐2, cleaved caspase‐3, cleaved caspase‐8, cleaved caspase‐9, and GAPDH in bEnd.3 cells after stretch injury transfected with siRNAs. GAPDH was used as the control for protein loading. n≥3/group (E) Cells apoptosis was analyzed through flow cytometry assay by Annexin V‐FITC/PI dual staining. Cells transferred with siRNA control were shown as Controls. Cells without stretch injury were shown as Blank. Quantitative analysis of (E), n = 3/group. Data were presented as means ± SEMs. Student's t test., ***p < 0.001; **p < 0.01; *p < 0.05 vs. siRNA control group.

The BBB is composed of brain microvascular ECs, a basement membrane, pericytes, and astrocytes [16]. Microvascular ECs play a critical role in BBB function. We next investigated the effect of GPR124 on ECs after TBI. As GPR124 expression in uninjured ECs was extremely low and nearly undetectable (Figure S3A)—consistent with our previous findings in human brain tissue—we first applied stretch injury to the cells before introducing any intervention (Figure 4A).

Multiple siRNAs targeting GPR124 were designed, and their knockdown efficiency was validated using qRT‐PCR and WB (Figure S3B,C, Figure 4B,C). Compared with the siRNA control group, siRNA1, 2, 4, and 5 significantly reduced GPR124 expression, whereas siRNA3 had no significant effect, and siRNA6 failed to achieve knockdown. Therefore, we selected siRNA1, 2, 4, and 5 for subsequent experiments. We then assessed the expression of tight junction proteins claudin‐5, occludin, and ZO‐1 in ECs (Figure 4D). WB results demonstrated that GPR124 knockdown reduced the expression of these tight junction proteins in bEnd.3 cells following stretch injury. These findings suggest that GPR124 enhances the expression of tight junction proteins and thereby mitigates BBB disruption both in vivo and in vitro.

**FIGURE 5:**
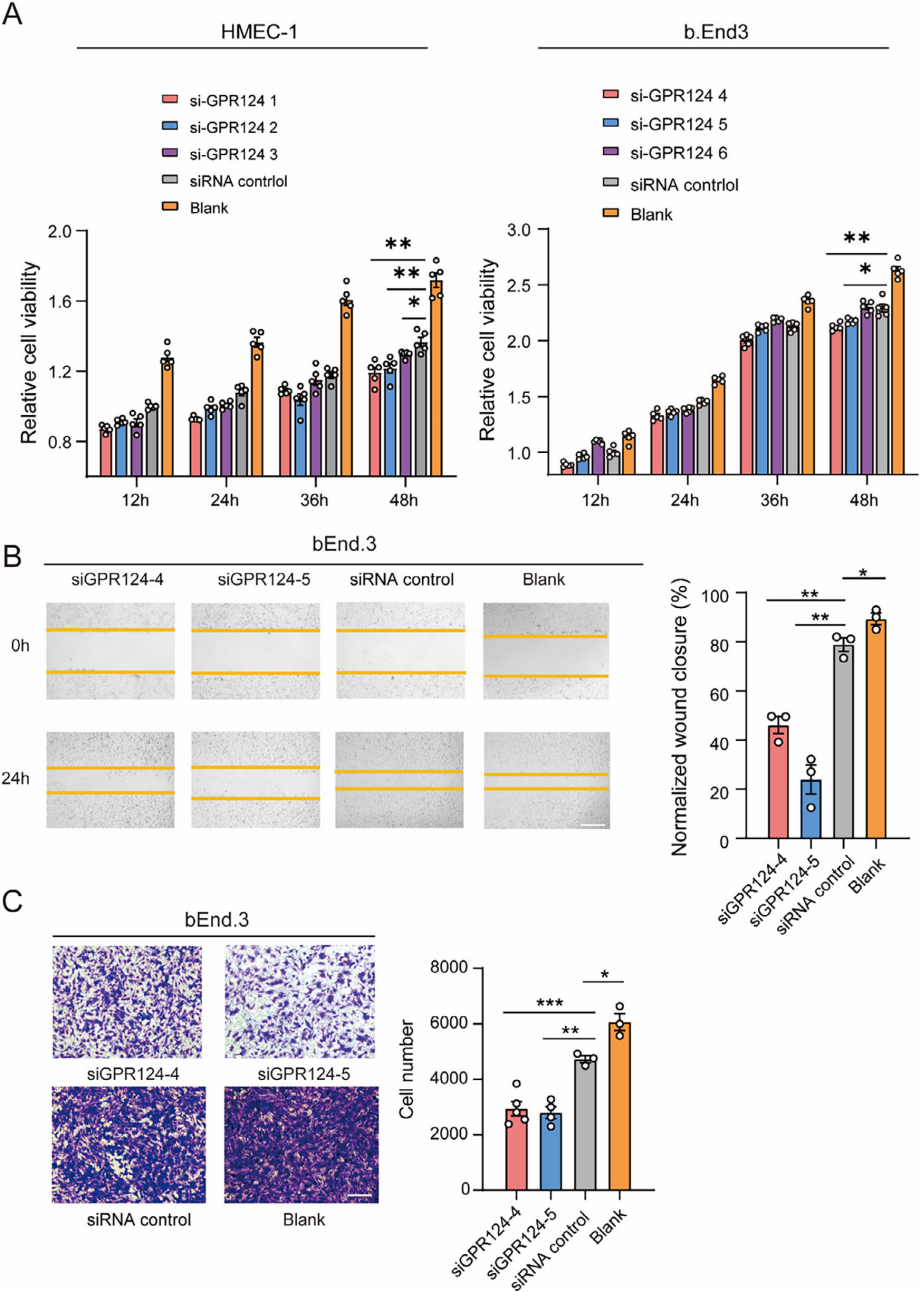
GPR124 promoted the proliferation and migration of endothelial cells after stretch injury in vitro. (A) CCK8 evaluated the effect of GPR124 knockdown on cell proliferation in HMEC‐1 and bEnd.3 cells after stretch injury. Scale bar,100 µm. n = 5/group. The cell viability result was normalized to stretch injury cells transferred with siRNA control for 12 h. Wound‐healing (B) and transwell assay (C)and to analyze the effect of siGPR124‐4, siGPR124‐5, siRNA control, and cells without stretch injury (Blank) on cell migration ability in bEnd.3 cells after stretch injury. Scale bar, 200 µm. n = 3/group. Quantitative analysis of B and C. Data were presented as means ± SEMs. Student's t test., ***p < 0.001; **p < 0.01; *p < 0.05 vs. siRNA control group.

In addition to tight junctions, ECs function plays a key role in maintaining the integrity of the BBB. First, we evaluated the effect of GPR124 on cell apoptosis using fluorescein isothiocyanate (FITC)/propidium iodide (PI) flow cytometry, which indicated that cell apoptosis increased significantly following GPR124 inhibition (Figure 4E). Cells without stretch injury showed lower rate of apoptosis than siRNA control group. Next, we investigated the mechanisms underlying GPR124‐mediated apoptosis. WB results revealed that the inhibition of GPR124 expression led to an increase in Bax expression, a decrease in Bcl‐2 expression, and a decrease in the Bcl‐2/Bax ratio, along with elevated levels of cleaved caspase−3, −8, and −9 (Figure 4D). These findings indicate that the apoptosis rate was higher in the GPR124 knockdown group than in the siRNA control group, and this apoptosis was mediated by Bax/Bcl‐2 signaling and the activation of caspases. Additionally, we performed a Cell Counting Kit‐8 (CCK‐8) assay in bEnd.3 and HMEC‐1 cells following stretch injury. Cell growth in the GPR124 knockdown group was significantly slower than that in the siRNA control group at 48 h, indicating that GPR124 suppression inhibits cell proliferation (Figure 5A). Subsequently, we performed wound healing and transwell migration assays. The wound closure area and number of migrated cells were reduced in the GPR124 knockdown group compared to those in the siRNA control group, indicating that inhibiting GPR124 expression significantly impaired the migratory ability of ECs (Figure 5B,C).

In addition, compared with the control group without injury(blank), the siRNA control group with stretch injury showed increased expression of apoptotic molecules (Figure S3D), a higher proportion of apoptotic cells (Figure 4E), decreased expression of tight junction proteins (Figure S3D), and reduced migration ability (Figure 5B,C), indicating that stretch injury was more effective than the damage in the sham group.

### 2.6 GPR124 Acts Through the Wnt/β‐Catenin Signaling Pathway

**FIGURE 6:**
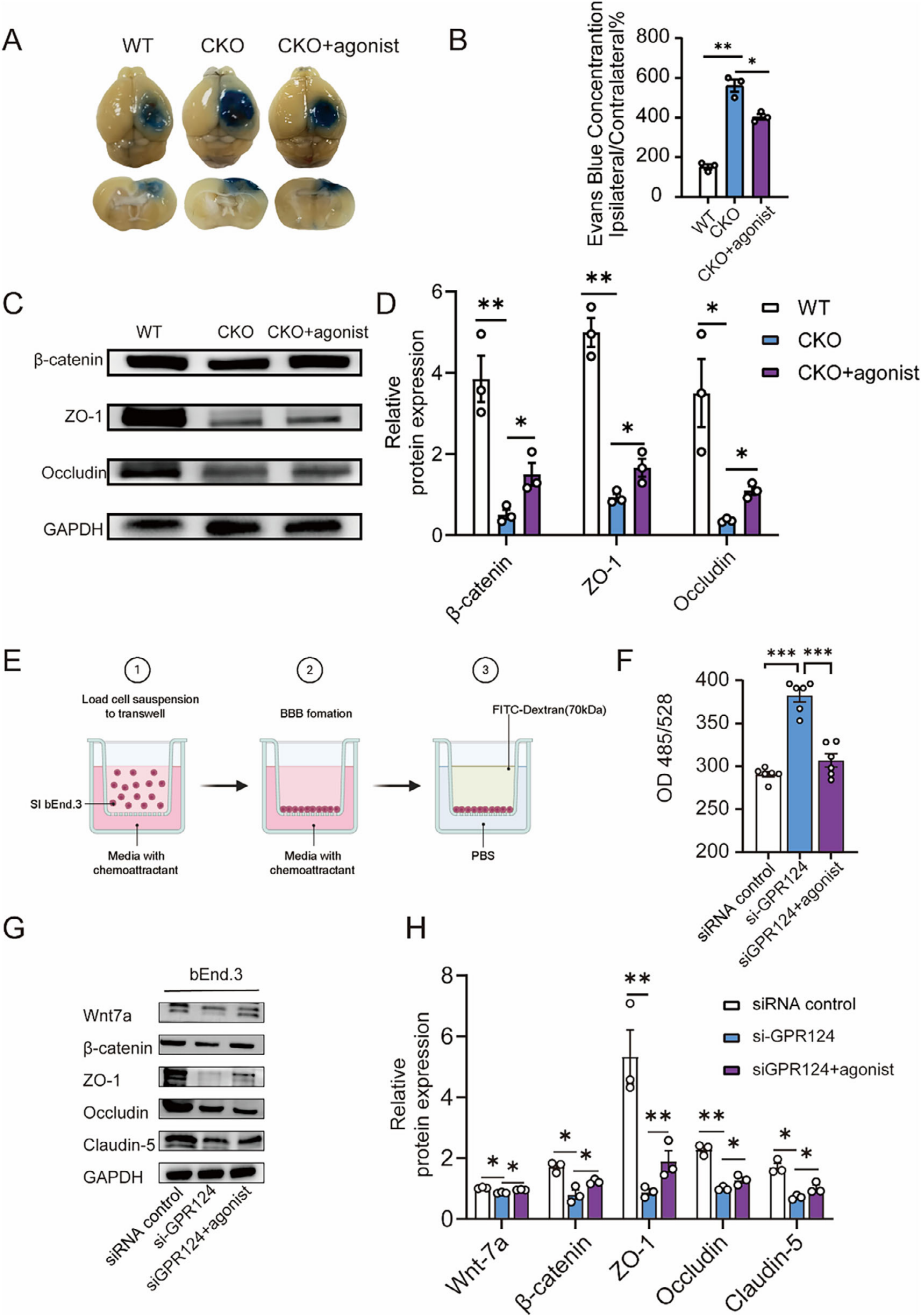
GPR124 acts through the Wnt/β‐catenin signaling pathway. (A) Representative image of EB extravasation in wild‐type, ECs‐special GPR124 conditional knockout (CKO), and CKO with Wnt signaling agonist group post‐insult (blue areas indicated the dye extravasation). (B) Statistical results of Figure A.CKO group was used as the control. n = 3/group. (C) Representative western blot results and quantitative analysis (D) of β‐catenin, ZO‐1, occludin, and GAPDH in WT, CKO mice, and CKO+agonist mice. GAPDH was used as the control for protein loading. The CKO group was used as the control, n = 3/group. (E) Schematic diagram of the cell stretch injury model in vitro BBB model. (F) Fluorescence analysis of the lower transwell chamber medium of siRNA control, siGPR124, and siGPR124+agonist. The siGPR124 group was used as the control, n = 5/group. (G) Representative western blot results and quantitative analysis (H) of Wnt7a, β‐catenin, ZO‐1, Occludin, Claudin‐5 and GAPDH in siRNA control, siGPR124, and siGPR124 + agonist group. GAPDH was used as the control for protein loading, siGPR124 was used as the control, n = 3/group. Data were presented as means ± SEMs. Student's t test., ***p < 0.001; **p < 0.01; *p < 0.05 vs. control group.

Previous studies have identified GPR124 as a co‐receptor of Wnt proteins, facilitating signal transduction into the cell [17]. To investigate whether this pathway mediates GPR124's protective effects, we intraperitoneally injected CHIR99021, a Wnt signaling agonist, into CKO mice 48 h prior to day 3 post‐TBI. The EB leakage assay demonstrated that the agonist‐treated group exhibited reduced EB leakage compared to the untreated control group (Figure 6A,B). Concurrently, we collected brain tissue for WB, which revealed higher expression levels of tight junction proteins ZO‐1 and Occludin in the agonist group (Figure 6C,D). Next, we constructed an in vitro BBB model (Figure 6E). Fluorescence analysis of the lower transwell chamber medium demonstrated that GPR124 suppression led to increased FITC‐dextran leakage and impaired in vitro BBB integrity, whereas the addition of agonists reversed this effect (Figure 6F). Finally, WB of EC samples confirmed that GPR124 knockdown reduced the expression of tight junction proteins, and the Wnt agonist was able to restore their levels (Figure 6G,H).

These in vivo and in vitro results indicate that knockdown of GPR124 compromises BBB integrity, and this effect is mediated through the Wnt/β‐catenin signaling pathway.

### 2.7 GPR124 binds to FGFBP1 to Activate the Wnt/β‐Catenin Signaling Pathway

**FIGURE 7:**
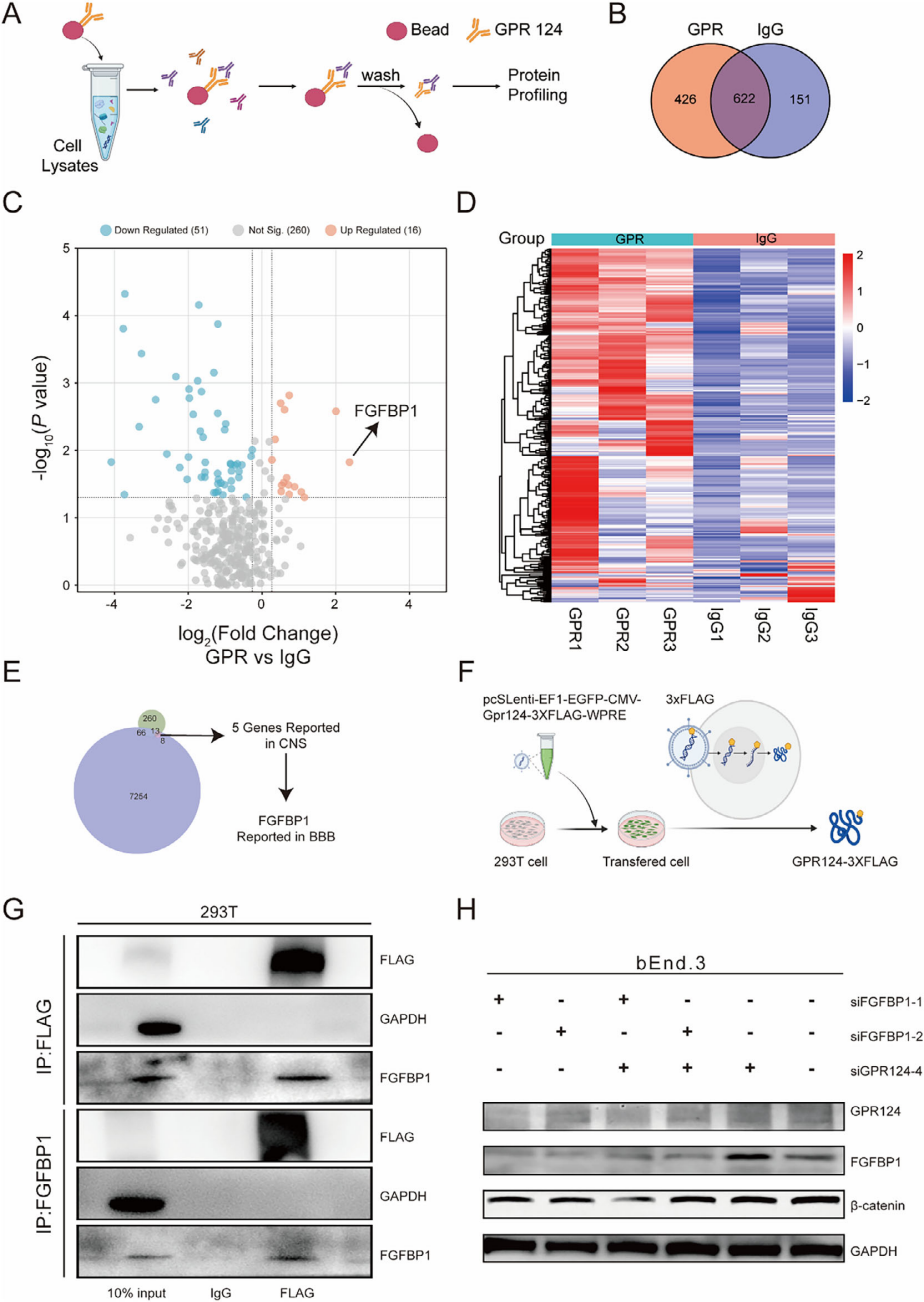
GPR124 can further exert its protective effect by binding to FGFBP1. (A) Schematic diagram of screening proteins that bind to GPR124. After the GPR124 antibody and magnetic beads are incubated and combined, the cell lysate is added. GPR124 binds to the proteins in the cells and then eluted, and the eluted proteins are detected by protein spectrum. (B) Venn diagram of mass spectrometry results. By analyzing the mass spectrometry results, proteins with increased binding rate in the GPR group compared with the IgG group and proteins with significantly reduced binding rate with the IgG group were screened out, thereby excluding nonspecific binding proteins. (C,D). (E) Elevating 3 days after TBI in single‐cell sequencing crossed with selected in C, D. Blue: genes increase in 3 days after TBI in single‐cell sequence. Green: proteins arise in the GPR124 group vs. IgG. Pink: proteins had been reported before. (F) Schematic diagram of overexpression of GPR124 and CO‐IP with FGFBP1 in 293T cells. (G) Representative CO‐IP western blot results. FLAG‐GPR124 pulldown followed by FLAG, FGFBP1, and GAPDH blotting. Then FGFBP1 pulldown was followed by FLAG, FGFBP1, and GAPDH blotting. (H) Representative western blot results of GPR124, FGFBP1, β‐catenin, and GAPDH after transfection of siGPR124 or siFGFBP1 in bEnd.3 cells after stretch injury.

To investigate the mechanism by which GPR124 functions, we employed co‐immunoprecipitation (Co‐IP) in 293T cells to isolate proteins that interact with GPR124, followed by mass spectrometry analysis (Figure 7A). From the mass spectrometry results, we identified proteins that were significantly enriched in the GPR124 group compared to the IgG control group (Figure 7B–D). We then cross‐referenced these proteins with those upregulated 3 d post‐TBI in our single‐cell sequencing dataset, resulting in the identification of 13 candidate proteins (Figure 7E). Based on existing literature, we focused on FGFBP1—a secreted extracellular matrix protein known to mediate neurovascular unit interactions and maintain Wnt/β‐catenin signaling activity in ECs.

Given the low basal expression of GPR124 in cells, we constructed a lentivirus vector containing a 3×FLAG tag to overexpress GPR124 in 293T cells. Co‐IP experiments using anti‐FGFBP1 and anti‐FLAG antibodies confirmed a physical interaction between GPR124 and FGFBP1 (Figure 7F,G). To further assess the functional relationship, we transfected with siRNA targeting GPR124 (si‐GPR124), FGFBP1 (si‐FGFBP1), or both, and the expression levels of GPR124, FGFBP1, and β‐catenin were assessed by WB. Simultaneous knockdown of GPR124 and FGFBP1 more significantly reduced β‐catenin expression than knockdown of either GPR124 or FGFBP1, indicating that the interaction between GPR124 and FGFBP1 positively regulates the Wnt/β‐catenin signaling pathway (Figure 7H).

## 3 Discussion

This study demonstrated that GPR124 is a novel post‐TBI protective factor that promotes neurological recovery by regulating the Wnt/β‐catenin signaling pathway, which plays a key role in BBB repair in an experimental TBI model. Mechanistically, GPR124 not only modulated the expression of tight junction proteins after TBI but also enhanced EC function by promoting proliferation, migration, and anti‐apoptotic activity. Notably, the reduction in cerebral hemorrhage resulting from these processes further contributed to improved neurological recovery. These findings suggest that GPR124 may serve as a potential therapeutic target for reducing cerebral hemorrhage and improving neurological outcomes following TBI.

TBI is a leading cause of death in young individuals, and its treatment remains challenging owing to the complex pathophysiological changes that occur in brain tissue following injury [4]. TBI is categorized into primary and secondary injuries based on underlying pathophysiological mechanisms [18]. Secondary injury is often considered more severe and can lead to both acute and chronic cellular and organ damage [19]. Interventions aimed at mitigating secondary injury have the potential to improve clinical outcomes. The secondary injury results from a series of cellular and molecular events in which the BBB plays a critical role as a protective barrier between the circulatory system and brain tissue [20].

Various studies have demonstrated that molecular changes before and after TBI are primarily associated with secondary injury [21, 22, 23]. In our study, we observed that GPR124 levels were elevated in human TBI brain tissue samples compared with those in ICH brain tissue. Next, in brain tissue from mice with CCI, immunofluorescence co‐staining for GPR124 and CD31 revealed that GPR124 expression increased during the acute phase of TBI and was localized to ECs. These findings strongly suggested that GPR124 plays a role in secondary injury after TBI and is associated with BBB function.

TBI‐mediated secondary injury impairs neurological recovery [24]. To investigate the impact of elevated GPR124, we generated EC‐specific GPR124 CKO mice and conducted behavioral experiments. Motor, sensory, balance, and reflex functions were assessed using the mNSS, hanging wire, and rotarod tests [25]. Muscle strength was measured using the grip strength and hanging wire tests, while spatial memory and cognitive function were evaluated using the Y‐maze test [26]. Notably, EC‐specific CKO of GPR124 led to worsened neurological performance in mice following TBI.

In addition, our experiment showed that at later stages of TBI (e.g., 14 d post‐TBI), neurological function was improved in mice with intact GPR124 compared to that of CKO mice, suggesting that GPR124 may be involved in a new repair mechanism during the long‐term recovery phase following TBI. We hypothesize that in the early stage after TBI, upregulation of GPR124 contributes to BBB repair, restoring its barrier function more rapidly, thereby isolating harmful substances such as inflammatory factors and reestablishing nutrient delivery. This creates a more favorable environment for subsequent neurological recovery.

Disruption of the BBB alters the microenvironment necessary for normal neuronal function and contributes to secondary injury [27, 28]. Moreover, BBB disruption and dysfunction are common in various CNS pathologies, such as TBI, Alzheimer's disease, stroke, epilepsy, brain tumors, and multiple sclerosis [29, 30, 31]. In this study, we observed that EC‐specific CKO of GPR124 resulted in a larger area of brain hemorrhage following TBI, with slower resolution of the bleeding over time. EB, a large molecule that cannot penetrate an intact BBB, is widely used to assess BBB permeability. We found that EC‐specific CKO of GPR124 led to more severe BBB leakage during the early stages of TBI. The precise regulation of the BBB integrity is attributed to its complex anatomical organization, which includes ECs, tight junctions, the basement membrane, pericytes, and astrocytic end‐feet, arranged from the vascular lumen to the brain parenchyma [32]. ECs are aligned along the basement membrane, and their surfaces are sealed by tight junctions, thereby limiting paracellular diffusion and playing a key role in maintaining BBB integrity [33]. IHC of early TBI sections revealed that tight junction proteins exhibited disrupted expression in ECs of EC‐specific GPR124 CKO mice. Taken together, these findings suggest that GPR124 mitigates BBB disruption.

The primary structural and functional component of the BBB is the EC. To simulate TBI conditions in vitro, we used an SI device and performed functional evaluation assays on the b.End3 cell line following SI. Cell proliferation was assessed using the CCK‐8 assay, apoptosis was measured via FITC/PI flow cytometry, and cell migration was evaluated using both transwell and wound‐healing assays [34]. Notably, when GPR124 expression was suppressed, cell growth and migration were impaired, apoptosis increased, and the expression of tight junction proteins decreased, all of which hinder the recovery of EC function after TBI. These findings suggest that GPR124 plays a protective role in maintaining EC function following TBI, thereby preserving BBB integrity and ultimately contributing to improved outcomes.

Bcl‐2 acts at the mitochondria level to inhibit cytochrome c release and suppress caspase activation, whereas Bax promotes cytochrome c release and apoptotic signaling [35]. The Bcl‐2/Bax ratio plays a critical role in determining cell fate. In this study, GPR124 inhibition led to increased Bax expression and decreased Bcl‐2 expression, resulting in a significant increase in the Bax/Bcl‐2 ratio, which may favor pro‐apoptotic signaling. Additionally, we observed an increase in cleaved caspase−3, −8, and −9, further confirming activation of the apoptotic pathway. These findings help clarify the molecular mechanisms underlying EC apoptosis following GPR124 inhibition.

Previous studies have frequently identified GPR124 as a co‐receptor of Wnt, acting in conjunction with Wnt receptors to transduce extracellular signals into the cell and promote the β‐catenin activation in the nucleus [10, 17]. However, few studies have explored the specific proteins that interact with GPR124. In this study, we used Co‐IP combined with proteomic analysis to screen for and confirm an interaction between GPR124 and FGFBP1. We also demonstrated their synergistic activation of Wnt/β‐catenin signaling, as shown by the enhanced inhibition of β‐catenin expression when both are simultaneously knocked down. To the best of our knowledge, this is the first study to investigate GPR124‐interacting proteins, offering a new insight into the molecular mechanisms by which GPR124 activates the Wnt pathway.

Despite these advances, this study has certain limitations. Although we did not detect the localization of GPR124 in other cell types within the brain, we cannot rule out the possibility that GPR124 may influence other components of the BBB, such as astrocytes or pericytes—an area that warrants further investigation. Additionally, although we demonstrated an interaction between GPR124 and FGFBP‐1, the molecular mechanism underlying this interaction remains uncharacterized and should be the focus of future studies. Finally, owing to the clinical characteristics of TBI, only patients with severe injuries undergo surgical intervention, allowing for tissue sample collection. Patients with moderate or mild TBI are generally treated conservatively, and their tissue samples are not available. As a result, we were unable to correlate GPR124 expression with TBI severity. This limitation may be addressed in future studies through the use of alternative biological samples.

## 4 Conclusion

In summary, building upon prior studies that emphasized the role of GPR124 in cerebrovascular development and BBB maintenance during embryogenesis, as well as its involvement in pathologies such as brain tumors and stroke, our study demonstrates that GPR124 is essential for preserving EC function and maintaining BBB integrity after TBI. Moreover, we show that GPR124 facilitates neurological recovery post‐TBI, primarily through the activation of the Wnt/β‐catenin signaling pathway. These findings provide new mechanistic insights into the role of GPR124 in CNS injury and highlight its therapeutic potential for improving outcomes following TBI.

## 5 Materials and methods

### 5.1 Human Brain Tissue Samples

The present study was conducted in accordance with the Declaration of Helsinki and was approved by the Research Ethics Board of Shanghai Sixth People's Hospital (2022‐KY‐13(K)). Written informed consent was obtained from all individuals who were included in the study. TBI patients were diagnosed according to the World Health Organization criteria. The clinical specimens of damaged brain tissue were taken from 4 patients who were operated on in the neurosurgical emergency department of Sixth People's Hospital. The experimental group was selected from patients with severe cerebral contusion, accompanied by malignant intracranial hypertension, who required internal decompression surgery, and the brain tissues cleared during the operation. The control group was selected from patients with deep cerebral hemorrhage, and the brain tissues on the hematoma path was cleared during the operation.

### 5.2 Single‐Cell RNA Sequencing Data Sets and Analysis

#### 5.2.1 Quality Control and Unsupervised Clustering

Unsupervised clustering of single cells was performed using Seurat R package with the above modified matrix as input. Before clustering the cells, we preprocessed the data of each sample as following steps. First, cells with detected genes less than 200 or genes expressed in less than three cells were removed. To obtain more reliable data, we further filtered cells with more rigorous criteria, i.e., cells with less than 500 genes or more than 5% of organelle genes (mitochondria genes) were excluded. The retained matrix was normalized using “NormalizeData” function with a global‐scaling normalization method (normalization.method = “LogNormalize”) in Seurat, in which the read counts of each gene in a given cell were divided by the total valid read counts of that cell and then multiplied by the scale factor (scale.factor = 10,000) before natural‐log transformed. After that, highly variable genes (HVGs) were calculated using the function “FindVariableGenes” (selection.method = “vst”, nfeatures = 3000). To avoid the dominant effect of highly‐expressed genes during analyses, a linear transformation was applied to scale the expression data using the “ScaleData” function, followed by the linear dimensional reduction using the function “RunPCA”. Finally, a K‐nearest neighbor (KNN) graph‐based approach implemented in functions “FindNeighbors” and “FindClusters” was employed to cluster all the retained cells into groups. Cell clustering results were visualized using both uniform manifold approximation and projection (UMAP). The DoubletFinder R package was imported to predicted the possible doublet cells of each tissue and remove the doublet cells of the data before subsequent analysis.

#### 5.2.2 Dentification of Cluster‐Specific Differentially Expressed Genes

To define feature genes for each cell cluster, differentially expressed genes (DEGs) between each cell cluster and all other cell clusters were detected using the “FindAllMarkers” function through the non‐parametric Wilcoxon Rank Sum test with only.pos = “TRUE”, logfc.threshold = 0.25 and min.pct = 0.25. The logfc.threshold parameter is the cutoff of expression fold change to test whether a gene was identified as differentially expressed in a certain cell cluster. The min.pct is the minimum percentage of a potential DEG expressed in all the cells.

### 5.3 Cell Type Annotation

To annotate the cell types for all clusters retrieved in brain tissues, we compiled known marker genes from previous neurobiological studies and recent single‐cell transcriptomic investigations. Each cluster's cell type was initially defined using well‐established marker genes that have been published.

In cases where specific clusters lacked well‐known marker gene support, we leveraged cluster‐enriched genes identified in previous single‐cell RNA sequencing (scRNA‐seq) studies to facilitate the annotation process. Notably, certain cell types representing specific developmental stages within the brain were underrepresented, complicating their clustering and identification in isolated tissue samples. To mitigate this challenge, we identified these cell types by aggregating them within clusters that corresponded to their specific identities in an integrated dataset encompassing multiple developmental stages of brain tissue.

Given the advantages of single‐nucleus RNA sequencing (snRNA‐seq) in capturing transcriptional diversity among different clusters within a single cell type, we maintained multiple clusters for each cell type, aligning with the majority of previously published studies in the field.

### 5.4 Animals

Wild‐type mice and EC‐specific conditional knockout (CKO) GPR124 mice were used in this study, aged 8– 10 weeks and weighing 23 ± 3 g were provided by the Shanghai Model Organisms Center, Inc.40 wild mice were assigned to 2 groups (total n = 40): (A) a sham‐operated group, in which the mice were subjected to surgery only; (B) a CCI model group, in which the mice received CCI. All animals were housed under a 12 h light/dark cycle at a constant temperature of 23°C with ad libitum access to food and water. All the experimental procedures were approved by the Ethics Committee of the Shanghai Jiao Tong University (Permission Number:2023022). The mouse experiments were performed according to the Guidelines for the Care and Use of Laboratory Animals from the National Institutes of Health.

The Gpr124^flox/flox^ allele was generated and crossed with the tamoxifen‐inducible endothelial driver Cdh5‐CreER on the C57BL/6 background to generate Gpr124^flox/flox^; Cdh5‐CreER^+/wt^ mice (termed CKO mice) and Gpr124^flox/flox^; Cdh5‐CreER^wt/wt^ mice (termed control mice). The Gpr124^flox/flox^ and CreER^+/wt^ mice did not show any differences from the wild‐type mice. The efficiency and specificity of Gpr124 gene knockout in the brain ECs of the CKO mice were confirmed in this study (Figure S4A–D). To induce Gpr124 deletion, 11 CKO mice and 11 control mice aged 7– 8 weeks were treated with tamoxifen (2 mg/10 g body weight, in corn oil) through an oral feeding needle every day for 5 days. The tamoxifen dosage was based on a previous study [15]. The mice were allowed to recover from tamoxifen treatment‐related toxicity (washout) for at least 10 days before being subjected to any other surgical procedures or experiments.

### 5.5 Controlled Cortical Impact Model of Traumatic Brain Injury

The TBI model used in this study has been described in detail in our previous studies [36]. Specifically, after anesthesia with a combination of xylazine (10 mg/kg) and ketamine (75 mg/kg), mice were fixed in a stereotaxic frame (Stoelting, Wood Dale, IL, USA) and an electric heating blanket was placed under the body to maintain the core body temperature at 37.0±0.5°C. After disinfecting the surgical area with 75% alcohol, a 1.5–2 cm incision was made in the midline of the scalp with a scalpel. Afterward, a 4 mm trephine was carefully used to perform surgery in the center of the right parietal bone, between the bregma and lambdoid, and 1 mm lateral to the sagittal suture. During the skull drilling process, if the dura mater was ruptured, the mouse was excluded from the study. A contusion was performed using a CCI model device (PinPoint Precision Cortical Impactor PCI3000; Hatteras Instruments Inc., USA) perpendicular to the brain surface using a round steel impact probe (3 mm in diameter). The impact velocity was 1.5 m/s, the impact duration was 100 ms, and the impact depth was 1.5 mm. Afterward, the bone flap was closed with sterile bone wax, and the scalp was sutured with interrupted 6‐0 silk sutures. All animals were placed in a 37°C heated cage after surgery until they were fully mobile. Sham‐operated animals were operated the same as the TBI group except for CCI.

### 5.6 Brain Tissue Preparation

Mice were anesthetized on the days required for the experiment and perfused with 20 mL of phosphate‐buffered saline (PBS) followed by 15 mL of 4% paraformaldehyde (PFA) per mouse. Brain tissue was then removed, fixed overnight in 4% PFA, dehydrated in 30% sucrose until it sank to the bottom, and then treated with pre‐cooled isopentane and stored at −80°C. Subsequently, 30 µm coronal sections from −1.0 to −3.0 mm from bregma were collected in 24‐well plates containing PBS plus 0.05% sodium azide and stored at 4°C until use.

### 5.7 Measurement of Evans Blue Extravasation

EB is used to assess the permeability of the blood–brain barrier to macromolecules. Because serum albumin cannot cross the barrier and nearly all EB binds to albumin, neural tissue usually remains unstained [37]. When the BBB is compromised, albumin‐bound Evans blue enters the central nervous system. Three days after TBI, mice were injected with 0.1 mL of EB (2% saline, catalog number E2129, Millipore Sigma) into the jugular vein, placed on a heating pad for 2 h, and then perfused with 50 mL phosphate‐buffered saline through the left ventricle to remove the EB dye from the blood circulation. The hemisphere ipsilateral to the CCI was immediately separated from the remaining brain and weighed with a precise analytical balance. After weighing, the ipsilateral hemisphere was homogenized in 1 mL of solvent (3:1 trichloroacetic acid: ethanol) using an ultrasonic homogenizer (Sonics & Materials Inc., Newtown, CT, USA). After centrifugation (12000 × g, 20 min), the solvent was separated into 2 layers, and the supernatant was collected and transferred to a 96‐well plate. The absorbance of the supernatant at 610 nm was detected using a microplate reader (Biotek, Winooski, VT, USA). Each sample was read in triplicate, and EB concentration was calculated based on the absorbance and standard curve. Finally, EB concentration was expressed as EB content per gram of brain tissue weight.

### 5.8 Neurobehavioral Function Assessments

Neurobehavioral assessments were recorded at 1, 3, 5, 7, and 14 days after TBI by an investigator blinded to experimental design using the modified Neurological Severity Score (mNSS), Grid‐Walking Test, Rotarod Test (RotorRod), Y maze Test and Hanging Wire Test. The detailed criteria were shown in Supplementary Methods.

### 5.9 Immunofluorescence Staining

Brain sections were treated with −20°C precooled 100% methanol for 10 min after antigen retrieval, followed by blocking with 1% bovine serum albumin (BSA) for 1 h. Next, brain sections were incubated with primary antibodies at 4°C overnight, followed by incubation with fluorescently labeled secondary antibodies at 37°C for 1 h, and then stained with DAPI solution. Staining was visualized using LSM710 laser scanning. Confocal fluorescence microscopy was performed and images were acquired using a 363‐oil immersion lens (Carl Zeiss, Oberkochen, Germany). The antibodies shown are listed in Supplemental Table S1.

### 5.10 Cell Culture and Reagents

The murine brain EC line, bEnd.3 cells, the human kidney cells, 293T and the human microvascular EC, HMEC‐1 cells, were purchased from the American Type Culture Collection (ATCC, Manassas, VA, USA). The bEnd.3 cells and 293T were maintained in complete Dulbecco's modified Eagle medium (DMEM) supplemented with 10% heat‐inactivated fetal bovine serum (FBS) and 100 U/ml penicillin/streptomycin. The HMEC‐1 cells were cultured in MCDB131, with 10 ng/mL Epidermal Growth Factor, 1 µg/mL Hydrocortisone, 10 mM Glutamine, FBS to a final concentration of 10% and 100 U/mL penicillin/streptomycin. All the cells were incubated in a humidified incubator at 37°C in 5% (v/v) CO_2_. The details of reagents used in this study were shown in Supplemental Table S1.

The si‐GPR124 and control siRNA were designed and synthesized by GenePharma (Shanghai, China). Lipofectamine 3000 reagent was used as a transfection medium according to the manufacturer's guidelines. Oligonucleotide sequences were listed in Supplemental Table S2.

### 5.11 Mechanical Stretch Injury

TBI in vitro was simulated using mechanical stretch injury (SI) of bEnd.3 and HMEC‐1 cells. Cells were seeded onto BioFlexÒ 6‐well culture plates (Flexcell International Corp., Burlington, NC) coated with a collagen‐coated silicone rubber membrane. After incubating the cells overnight, biaxial SI was induced in the cells using the Cell Injury Controller II system (Virginia Commonwealth University, Richmond, VA). The instrument released a 50 ms burst of nitrogen gas, causing the silicone rubber membrane and adherent cells to deform downward by 7.5 mm, similar to the mechanical stress imposed on brain tissue by rotational acceleration and deceleration injuries.

### 5.12 Quantitative Reverse Transcription Polymerase Chain Reaction

Total RNA was extracted from tissues or cells using TRIzol Reagent according to the manufacturer's instructions. The concentration and purity of RNA samples were measured by Nanodrop 2000 instrument (Thermo Fisher Scientific, USA). For mRNA analysis, the reverse transcription reactions were performed using the High‐Capacity cDNA Reverse Transcription Kit and qRT‐PCR was performed using SYBR Green Real‐Time PCR Master Mixes. GAPDH was applied as an endogenous standard control for normalization. The relative quantification of RNA expression levels was analyzed using the 2^−ΔΔCT^ method. Primer sequences were listed in Supplemental Table S2.

### 5.13 Western Blotting Analysis

Tissue and cells were lysed in RIPA Lysis Buffer on ice. The total protein concentration was measured and quantified by BCA protein assay. The equal amounts of total protein from different samples were separated by SDS‐PAGE gels and transferred onto polyvinylidene fluoride (PVDF) membranes. The membranes were blocked with 5% non‐fat milk and incubated with specific primary antibodies, which were listed in Supplemental Table S2 at 4°C overnight. Then, the membranes were washed with 1×TBST and incubated with anti‐rabbit or anti‐mouse HRP‐conjugated secondary antibodies (1:5000, Cell Signaling Technology, USA) for 1.5 h at room temperature. The membranes were visualized by an enhanced chemiluminescence kit. GAPDH was used as an endogenous standard control for normalization. The images were analyzed by Image J Software (National Institutes of Health, Bethesda, MD, USA).

### 5.14 Cell Counting Kit‐8 Proliferation Assay

Cell proliferative capacity in different groups was measured by CCK‐8 reagent. A total of 3000 cells were seeded in each well of a 96‐well plate. Before the detection of the optional density at 450 nm by an automatic microplate reader (Bio‐Rad, USA), each wells were added with 10 µL of CCK‐8 reagent and incubated at 37°C for 1 h. Cell proliferation was recorded at different time points (12, 24, 36, and 48 h). Each experiment was performed in triplicate.

### 5.15 Wound‐Healing Assay

Cells were seeded in 6‐well plates and incubated with drugs for an additional 24 h until they reached about 90–95% confluence. Afterward, a vertical scratch wound was created in the cell monolayer of each well's central area using a 200ul pipette tip. Subsequently, cells were washed with 1×PBS twice and then cultured in the serum‐free DMEM medium. The wound closure photographs were taken at 0 and 24 h after injury. Cell migration was quantified following the equation: (0 h wound area−24 h wound area)/0 h wound area×100%. The images were analyzed by ImageJ Software. Each experiment was performed in triplicate.

### 5.16 Transwell Migration Assay

The transwell chambers (8 µm pore size, Corning, USA) were used to measure the abilities of cells’ invasion after transfection. After transfection with drugs for 48 h, the cells were washed with 1x PBS twice and resuspended in their corresponding serum‐free culture medium (1×10^6^ cells/mL). Then, 800 µL medium with 10% FBS was added to the bottom chamber and 100 µL of the suspension was seeded into the upper chamber. After incubation for 24 h at 37°C, the upper chambers were fixed with a 4% PFA for 30 min and stained with 1% crystal violet solution for 15 min. The images were analyzed by ImageJ Software. Three visual fields were randomly selected for manual counting. Each experiment was performed in triplicate.

### 5.17 Cell Apoptosis Assay

Cell apoptosis was detected by Annexin V‐FITC/propidium iodide (PI) kit and measured by flow cytometer (FACS Calibur, USA). After transfection with drugs for 48 h, the cells were digested by trypsin, washed with ice‐cold 1×PBS, and stained with 5 µL FITC and 5 µL PI in the dark for 15 min. The results were analyzed by FlowJo software (Tree Star, USA).

### 5.18 Isolation and Culture of Mouse Brain Microvascular Endothelial Cells

Mice were killed by cervical dislocation and immersed in 75% ethanol for 3–5 min for disinfection. Then the heads were decapitated and placed in a glass culture dish. After the cranial cavity was opened, the whole brain was taken out and placed in a glass culture dish containing cold HBSS to dissect and remove the cerebellum and diencephalon (including hippocampus). The cerebral hemispheres were then slowly rolled on dry filter paper to remove the pia mater and large blood vessels of the meninges and placed in a new glass culture dish containing cold HBSS. The white matter of the brain, residual large blood vessels and pia mater were removed with fine dissecting forceps, and the cerebral cortex was retained. After rinsing with HBSS solution for 3 times, 1 mL of DMEM was added, and the mice were cut into 1 mm3 pieces with iris scissors. Type II collagenase (1 mL/brain, 1 mL = 10 µL 100X stock solution + 990 µL DMEM/F12) was added, and the mixture was digested at 37°C for 1.5 h without visible tangible substances. At the same time, 1X hydrolyzed fish bladder collagen (HFB) was used for plating. Add an equal volume of complete medium to terminate digestion, centrifuge (1000 rpm, 5 min, room temperature), remove the supernatant, and keep the precipitate. Add 15 mL of 20% BSA to the precipitate for suspension, mix and centrifuge (1000 g, 20 min, 4°C), remove the middle and upper layers of nerve tissue and large blood vessels, and keep the bottom precipitate. Add 3 mL of 25% trypsin digestion solution for 20 min, add an equal volume of serum‐containing medium to terminate digestion, centrifuge (1000 rpm, 8 min, room temperature), remove the supernatant, add 2 mL of DMEM culture medium for suspension, and then layer on 12 mL of 50% Percoll (25 000 g, 60 min, 4°C) that has been centrifuged to form a continuous gradient. Centrifuge (1000 g, 10 min, 4°C), the white and yellow layer above the red blood cell layer near the bottom is the purified microvessel segment, aspirate it and rinse it twice with DMEM (centrifuge 1000 rpm, 5 min, room temperature), remove the supernatant, gently rinse the excess HFB in the 6‐well plate with PBS, rinse it twice. Add DMEM complete culture medium (containing 20% FBS) to suspend and inoculate it in a 6‐well plate (1.5 mL/culture dish, 1 culture dish/brain can be inoculated), place it in a 37°C, 5% CO_2_ incubator for static culture, change the medium after 12–24 h, and then change the medium every other day.

### 5.19 Statistical Analysis

Statistical analyses were performed using the GraphPad Prism 8.02 (GraphPad Software, USA). All data were presented as mean ± SEM. Two‐tailed Student's t‐tests and one‐way analysis of variance followed by Bonferroni's post hoc test were used to calculate statistical significance. The Pearson correlation coefficient was used to analyze the correlations. *p*‐value <0.05 was considered statistically significant.

## Author Contributions

CW and LC conducted the research and wrote the manuscript; GQ, YY, YL, XN, LW, ZL, and SW helped to complete the experiment; CW and YM performed the statistical analysis and edited the manuscript; JD, HT and HC guided the entire study and provided the supervision and final check. All authors read and approved the final manuscript.

## Conflicts of Interest

The authors declare no conflicts of interest.

## Supporting information




**Supporting File 1**: advs76107‐sup‐0001‐SuppMat.docx.

**Supporting File 2**: advs76107‐sup‐0002‐Explanation_FigureS1B.docx.

## Data Availability

The scRNAseq data have been deposited in Gene Expression Omnibus. (GSE302863). Cai L., et al., Data from “ALKBH5 Demethylates the m6A Modification of SOCS3 in Microglia/Macrophages and Alleviates Neuroinflammation after Brain Injury.” GEO. https://www.ncbi.nlm.nih.gov/geo/query/acc.cgi?acc = GSE302863. Deposited 17 July 2025.
