# Supplementary material for: GPR124 Alleviates Blood–Brain Barrier Disruption by Enhancing Microvascular Endothelial Function after Traumatic Brain Injury
Source: Adv Sci (Weinh). 2026 Jun 15;13(51):e01197. doi: 10.1002/advs.202501197 (PMC13336676; doi:10.1002/advs.202501197)
Supplement: Supplementary file 1 [file ADVS-13-e01197-s001.docx]

Full membrane with molecular weight marker

Original GAPDH blot image obtained from a separate exposure

Original GPR124 blot image obtained from a separate exposure
