# Supplementary material for: GPR124 Alleviates Blood–Brain Barrier Disruption by Enhancing Microvascular Endothelial Function after Traumatic Brain Injury
Source: Adv Sci (Weinh). 2026 Jun 15;13(51):e01197. doi: 10.1002/advs.202501197 (PMC13336676; doi:10.1002/advs.202501197)
Supplement: Supplementary file 2 [file ADVS-13-e01197-s002.docx]

**GPR124 alleviates blood-brain barrier disruption through improving the function of microvascular endothelial after traumatic brain injury**

*Chen Wang^1#^, Lin Cai^1#^, Qiuyuan Gong^1^, Yang Yang^1^, Yuqing Liang^1^, Xinyu Niu^1^，Lai Wei^1^, Ze Liu^2^, Shengju Wu^2^, Yinghui Men^2^, Yaohui Tang^2^, Jun Ding^1*^, Hengli Tian^1*^, Hao Chen^1*^*

**1. Supplementary figures**

Figure S1. GPR124 was up-expressed after TBI. **A** Representative fluorescence images dual staining of GPR124(green) and CD31(red) in human brain tissue of TBI and ICH groups. Cell nuclei were shown in blue (DAPI). **B** Representative western blot results of GPR124 and GAPDH in human brain tissue from TBI and ICH groups. ICH group was set as control, n≥3/group. **C** Quantitative analysis of western blot results of GPR124 in human brain tissue of TBI and ICH groups. GAPDH was used as the control for protein loading. ICH group was set as control, n≥3/group. **D** qRT-PCR analysis of GPR124 mRNA in human brain tissue of TBI and ICH groups. ICH group was set as control, n=3/group. **E** Representative fluorescence images for dual staining of GPR124(green) and CD31(red) in mouse brain tissue from sham group. Cell nuclei were shown in blue (DAPI). **F** Schematic diagram of CCI in mice using a stereotaxic apparatus. **G** Representative western blot results of GPR124 and GAPDH in mouse brain tissue from Sham, TBI at 1, 3, 5, 7 and 14 days post-TBI. GAPDH was used as the control for protein loading. ICH group was set as control, n≥3/group. **H** Quantitative analysis of western blot results of GPR124 in mouse brain tissue from Sham, And TBI groups at 1, 3, 5, 7 and 14 days post-TBI. GAPDH was used as the control for protein loading. Sham group was set as control, n=3/group. **I** qRT-PCR analysis of GPR124 mRNA in mouse brain tissue from Sham, and TBI groups at 1, 3, 5, 7 and 14 days post-TBI. Sham group was set as control, n≥3/group. Student t test. Data were shown as mean ± SEM. **P*<0.05, ***P*<0.01, ****P*<0.001 vs. control group.

.

Figure S2. The co-localization of GPR124 (green) and neurons, microglia (red) were further confirmed. Representative fluorescence images dual staining of GPR124 and NeuN (**A**), IBA-1 (**B**) in mouse brain tissue from TBI groups at 1, 3, 5, and 7 days post-insult. scale bar, 100 μm.

Figure S3. A Representative western blot results of GPR124 and GAPDH in HMEC-1 and bEnd.3 cells without stretch injury but transfected with siRNAs. GAPDH was used as the control for protein loading. SiRNA control group was set as control. Representative western blot results of GPR124 and GAPDH in HMEC-1 cell after stretch injury transfected with siRNAs. GAPDH was used as the control for protein loading. SiRNA control group was set as control. **B** Quantitative analysis of western blot results of GPR124 in HMEC-1cell after stretch injury transfected with siRNAs. GAPDH was used as the control for protein loading. SiRNA control group was set as control n=3/group. qRT-PCR analysis of GPR124 mRNA in HMEC-1 (**C**) cell after stretch injury transfected with siRNAs. **D** Representative western blot results of occludin,BCL-2,cleaved caspase8 and GAPDH in bEnd.3 cells without stretch injury and transfected with siRNAs after stretch injury and without stretch injury(Blank) . Quantitative analysis of western blot results of Occludin, BCL-2, cleaved caspase8 and GAPDH in bEnd.3 cells without stretch injury and transfected with siRNAs after stretch injury. GAPDH was used as the control for protein loading. SiRNA control group was set as control n=3/group. Student’t test. Data were shown as mean ± SEM. **P*<0.05, ***P*<0.01, ****P*<0.001 vs. control group.

.

Figure S4. Verification of the successful construction of GPR124 conditional knockout mice. **A** The co-localization of GPR124 and ECs was confirmed. Representative fluorescence images dual staining of GPR124(green) and CD31 (red) in mouse brain tissue from TBI groups at 3 days post-insult. Primers (**B**) and electropherograms (**C and D**) for gene identification. **E** Representative western blot results and quantitative analysis (**F**) of GPR124 and GAPDH mRNA in isolated brain microvascular endothelial cells. GAPDH was used as the control for protein loading. n=3/group. Student t test. Data were shown as mean ± SEM. **P*<0.05, ***P*<0.01, ****P*<0.001 vs. control group.

Figure S5 Tamoxifen alone did not influence BBB integrity or neurobehavioral outcomes.

**A** Representative image of EB extravasation in sham and sham with tamoxifen groups n=3/group. **B** Statistical results of Figure **A**. n=3/group. **C-G** Graphs showed five neurobehavioral baseline including mNSS scores (C), Grid-strength test (D), Rotarod test (E), Hanging Wire test (F) and Y maze test (G). n=8/group. Sham group was set as control group. n=8/group. Student t test. Data were shown as mean ± SEM. **P*<0.05, ***P*<0.01, ****P*<0.001 vs. control group.

**2. Supplementary methods**

**2.1 The modified neurological severity score**

The modified neurological severity score (mNSS) test was performed to evaluate the neurological deficits of each mouse at 1, 3, 7, and 14 days after CCI. Scoring details are as follows. Motor deficits: raising the mouse by the tail to evaluate forelimb flexion (0–3) and placing the mouse on the floor to evaluate gait (0–3). Balance deficits: placing the mouse on a balance beam to assess posture (0–6). Reflex deficits: testing pinna and corneal reflexes (0–2). A score of 0 indicates normal behavior, whereas a score of 14 indicates maximal deficit[1].

**2.2 The rotarod test**

The rotarod test was performed to evaluate motor coordination of the mice. Mice were trained for 72 hours (three sessions/day for 5 minutes/session) before CCI; there was at least a 15-minute interval between each session. The speed of the rod was increased from 0 to 20 rounds/minute on the first day to 0 to 30 rounds/minute on the second day and 0 to 40 rounds/minute on the third day. Mice that could not stay on the rod for at least 5 minutes were excluded from this test. On the 4^th^ day, CCI was performed; mice were subjected to the rotarod test before CCI and at 1, 3, 7, and 14 days after CCI. The latency (time taken until the mouse fell off the rod) was recorded to evaluate the motor coordination function.

**2.3 The grip strength test**

First, place the mouse or rat on a fixed platform and let them stretch out their front or hind paws naturally. Then fix the measuring device of the grip strength tester on the paws of the mouse or rat, let them hold the elastic metal strip and gradually pull the elastic metal strip outward until the mouse can no longer maintain the grip and the metal strip falls off from their paws.

During the test, the measuring device will record the maximum grip strength value and display it on the screen of the instrument. Through multiple tests, the average grip strength value of the mouse can be obtained, and then its muscle strength and nervous system function status can be evaluated.

**2.4 The** **hanging wire test**

Hanging Wire Test was to evaluate limb muscle function and motor coordination over time. A 55 cm wide 2 mm thick metallic wire was secured to two vertical stands. The wire should be tightly attached to the frame to avoid vibration or unwanted displacement of the wire. Mice were subjected to 180 seconds lasting hanging test, during which the “falling” scores were recorded. When mice fell from the wire, the “falling” scores were diminished by one respectively. A Kaplan-Meier-like curve could be created afterward.

**2.5 The Y-Maze test**

The Y-maze test is a behavioral test widely used in neuroscience and behavioral research, mainly used to evaluate the spatial memory and cognitive function of animals.

The device was a symmetrically closed maze with 3 arms, the angle between the arms was 120°, the length of the arms was 28 cm, the width of the maze channel was approximately 5 cm, and the height of the maze wall was approximately 10 cm. In the first phase, one arm was closed, and each mouse was placed in the center of the device and allowed to explore the two open arms freely for 10 min. The second experiment was started with all arms open one hour later. The mice were allowed to explore all three arms freely for 5 min. The time and number of entries for each arm were recorded.

**2.6 Reference**

[1]	J. Chen, P. R. Sanberg, Y. Li, L. Wang, M. Lu, A. E. Willing, J. Sanchez-Ramos, M. Chopp, Stroke 2001, 32 (11), 2682, <https://doi.org/10.1161/hs1101.098367>.

**3. Supplemental Table 1**

| **Supplemental Table 1. Reagents used in this study** | | |
| --- | --- | --- |
| Reagents | Company | Cat No. |
| Dulbecco’s Modified Eagle Medium (DMEM) | HyClone, USA | SH30243 |
| Fetal Bovine Serum (FBS) | Gibco, USA | 10100-147 |
| Penicillin/Streptomycin | Gibco, USA | 15070-063 |
| Trypsin/EDTA | Gibco, USA | 25200-056 |
| Phosphate Buffered Saline (PBS) | Meilunbio, China | MA0015 |
| Paraformaldehyde (PFA) | Sigma-Aldrich, USA | 158127 |
| Methanol | Sigma-Aldrich, USA | 179337 |
| Bovine Serum Albumin (BSA) | YEASEN, China | 36104ES25 |
| Goat Anti-Iba1 for IF | Novusbio, USA | NB100-1028 |
| Rabbit Anti-Neun for IF | Abcam, USA | ab177487 |
| Goat Anti-CD31 for IF | Bio-RAD, USA | AF3628 |
| Rabbit Anti-ZO-1 for IF and WB | Thermo Fisher, USA | 61-7300 |
| Rabbit Anti-Occludin for IF and WB | Thermo Fisher, USA | 40-4700 |
| DAPI | Thermo Fisher, USA | D1306 |
| AF488 Donkey anti-Rat | Thermo Fisher, USA | SA5-10026 |
| AF488 Donkey anti-Mouse | Thermo Fisher, USA | A-21202 |
| AF555 Donkey anti-Goat | Thermo Fisher, USA | A-21432 |
| AF488 Donkey anti-Rabbit | Thermo Fisher, USA | A-21206 |
| AF555 Donkey anti-Goat | Thermo Fisher, USA | A-21432 |
| Cell Counting Kit-8 (CCK-8) | Dojindo, Japan | CK04 |
| TRIzol™ reagent | Thermo Fisher, USA | 15596-026 |
| Hifair® Ⅱ 1st Strand cDNA Synthesis SuperMix for qPCR | YEASEN, China | 11123ES10 |
| Hieff® qPCR SYBR Green Master Mix | YEASEN, China | 11203ES03 |
| Total Nitric Oxide Assay Kit (Colorimetric) | YEASEN, China | 50107ES50 |
| PE Annexin V Apoptosis Detection Kit I | BD Biosciences, USA | 559763 |
| Cell Lysis Buffer | Beyotime, China | P0013J |
| Enhanced BCA Protein Assay Kit | Beyotime, China | P0010 |
| PVDF membranes | Millipore, USA | IPVH00010 |
| Anti-Cleaved Caspase-3 antibody | abcam | ab2302 |
| Cleaved Caspase-8 (Asp387) (D5B2) XP® Rabbit mAb (Mouse Specific) | Cell Signaling Technology, USA | 8592 |
| GAPDH (D16H11) XP® Rabbit mAb | Cell Signaling Technology, USA | 5174 |
| Cleaved Caspase-9 (Asp353) Antibody (Mouse Specific) | Cell Signaling Technology, USA | 9509 |
| Bax Antibody | Cell Signaling Technology, USA | 2772 |
| Bcl-2 (D17C4) Rabbit mAb (Mouse Preferred) | Cell Signaling Technology, USA | 3498 |
| Mouse/Rat CD31/PECAM-1 Antibody | Bio-RAD, USA | AF3628 |
| Lipofectamine™ 3000 Transfection Reagent | Thermo Fisher, USA | L3000075 |
| Opti-MEM™ I Reduced Serum Medium | Thermo Fisher, USA | 31985070 |
|  |  |  |
| MCDB131 (without L-Glutamine） | Thermo Fisher, USA | 10372019 |
| Epidermal Growth Factor | Thermo Fisher, USA | PHG0314 |
| Hydrocortisone | Sigma-Aldrich, USA | H0396 |
| L-Glutamine | Thermo Fisher, USA | A2916801 |
| Anti-GPCR GPR124 antibody | Abcam, USA | ab198817 |

**4. Supplemental Table 2**

| Supplemental Table 2. Nucleotide Sequences Used in This Paper | | |
| --- | --- | --- |
| Nucleotide Sequences | Forward primer (5' to 3') | Reverse primer (5' to 3') |
| GAPDH for qRT PCR | GAGTCAACGGATTTGGTCGT | TGTGGTCATGAGTCCTTCCA |
| GPR124 for qRT PCR | TCACGCTCACCAACTACCAAATG | CCTCCAGCAATCAAGTAGAAC |
| siGPR124-1 for transfection | GAC UAA ACA UAU CUG GAA A TT | UUU CCA GAU AUG UUU AGU C TT |
| siGPR124-2 for transfection | CCA GCA AGA AGG UGG AGA U TT | A UCU CCA CCU UCU UGC UGG TT |
| siGPR124-3 for transfection | CAG UCA ACA UCC ACA ACU A TT | U AGU UGU GGA UGU UGA CUG TT |
| siGPR124-4 for transfection | GGA CUU UGG UAC UGA GUU U TT | A AAC UCA GUA CCA AAG UCC TT |
| siGPR124-5 for transfection | GCA AGA AGG UGG AGA UAG U TT | A CUA UCU CCA CCU UCU UGC TT |
| siGPR124-6 for transfection | CGCUCAACAUCCACAACUA TT | UAGUUGUGGAUGUUGACCG TT |
| siRNA contrlol for transfection | UUCUCCGAACGUGUCACGUTT | ACGUGACACGUUCGGAGAATT |
| siFgfbp1-1 for transfection | UCUUUGUCGUGUUUAAGGCAG | GCCUUAAACACGACAAAGACC |
| siFgfbp1-2 for transfection | AACAUGUUGAGGAAGAAUGUG | CAUUCUUCCUCAACAUGUUAC |
