# Supplementary material for: GPR124 Alleviates Blood–Brain Barrier Disruption by Enhancing Microvascular Endothelial Function after Traumatic Brain Injury
Source: Adv Sci (Weinh). 2026 Jun 15:e01197. Online ahead of print. doi: 10.1002/advs.202501197 (PMC13336676; doi:10.1002/advs.202501197)
Supplement: Supplementary file 2 — Supporting File 2: advs76107‐sup‐0002‐Explanation_FigureS1B.docx. [file ADVS-9999-e01197-s002.docx]

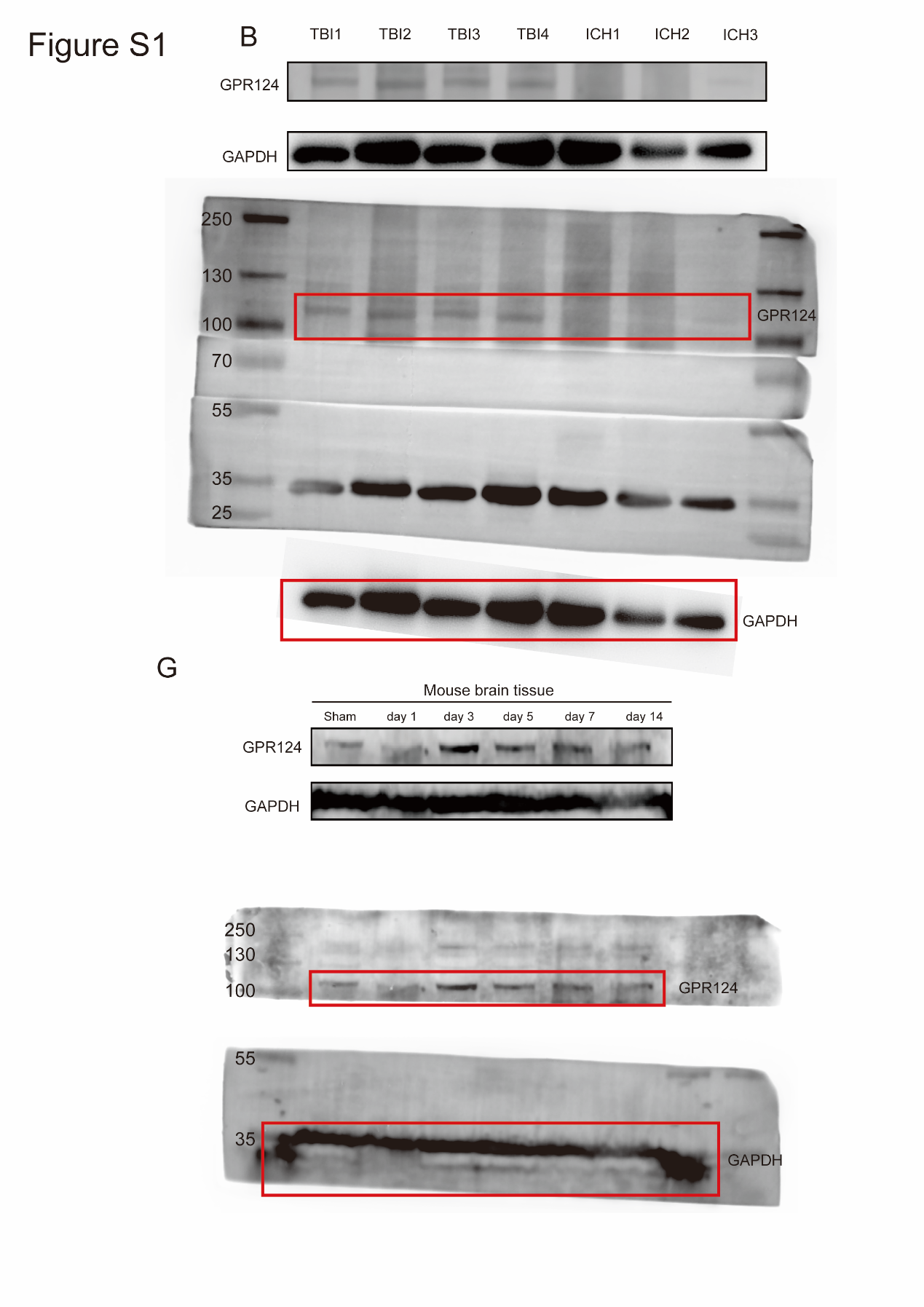


Full membrane with molecular weight marker


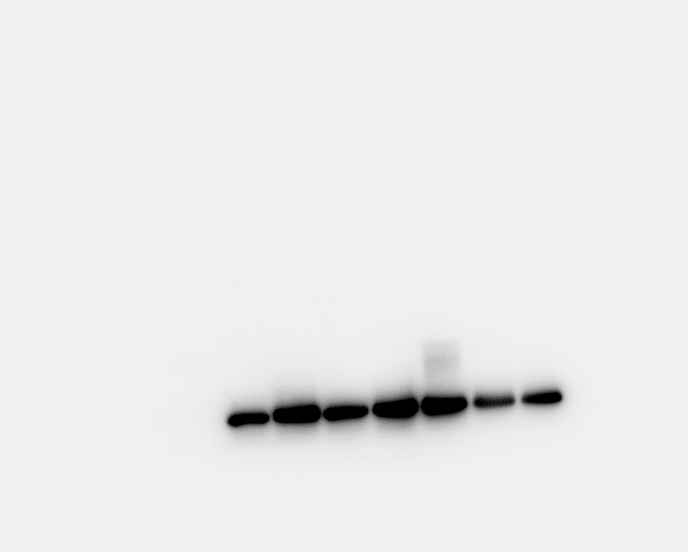


Original GAPDH blot image obtained from a separate exposure





Original GPR124 blot image obtained from a separate exposure
